# An Exploration of the Relationships Between Mental Wellbeing and Religion Amongst Students Attending Post-Primary Schools in Ireland

**DOI:** 10.1007/s10943-025-02307-5

**Published:** 2025-04-22

**Authors:** Lydia Mannion, Maurice Harmon, Trevor O’Brien

**Affiliations:** Mary Immaculate College, South Circular Road, Limerick, Ireland

**Keywords:** Mental wellbeing, Religion, Adolescents, Post-primary schools, Ireland

## Abstract

The current study aimed to explore the relationships between mental wellbeing and religion amongst adolescent students attending post-primary/second-level schools in Ireland. Adolescent student participants (*N* = 7) attending post-primary schools in Ireland completed online, semi-structured interviews on their perceptions regarding the relationship between mental wellbeing and religion. Reflexive thematic analysis from the critical realist perspective was employed to analyse the qualitative data. The results revealed a positive relationship between mental wellbeing and religion amongst participants. Specifically, aspects of religiosity, such as personal prayer, holding religious beliefs and religious practice were mentioned by participants as being beneficial for their mental wellbeing. Similarly, the practice of positive religious coping methods, such as reading of scripture for strength, having a relationship with God, and interpret negative life events in light of religion, had a positive impact on the mental wellbeing of participants. The findings demonstrate that for the participants in this small-scale study, religion has the potential to positively impact their mental wellbeing. Implications for areas of investigation in future research, curriculum and religious adolescents, as well as the adults who work with them in school and community settings, are discussed.

## Introduction

Within the context of Ireland, the religious demographic has transformed drastically over the past century (Inglis, 2017). In the twenty-five years between 1991 and 2016, census data has shown significant increases in the population of Ireland who do not identify as Roman Catholic (hereafter ‘Catholic’), particularly amongst those affiliated with other world religions and those who describe themselves as having no religion (Central Statistics Office [CSO], 1991; 2016). Despite the relative secularisation of Irish society in recent years, religion remains part of the lives of many adolescents in Ireland, perhaps as a result of Catholicism being culturally embedded into Irish society for centuries (Cullen, 2019).

A growing body of international research using quantitative methodologies demonstrates the potential of religion to affect the mental health of adolescents, either positively or negatively (Wong et al., 2006). For example, previous research has demonstrated that having a relationship with a religion is associated with an enhanced sense of purpose in life (Francis, 2013), greater levels of happiness (Holder, Coleman, Krupa and Krupa, 2016), more positive affect, higher self-esteem and greater satisfaction with life for adolescents (Abdel-Khalek, 2011, 2014; Davis III and Kiang, 2016). In addition, religion has been found to have a mitigating effect against anxiety (Abdel-Khalek, 2011) and depressive symptoms (Davis III and Kiang, 2016), as well as being a protective factor against the impact of racial stigmatisation for adolescents in ethnic minority groups (Butler-Barnes, Martin and Hope, 2018). Conversely, religion has also been linked to negative implications for adolescents’ mental health, including lower levels of happiness and a greater likelihood of experiencing depressive symptoms (Wenger, 2011). In addition, research has shown that adolescents’ religious attitudes can have the potential to be beneficial in terms of promoting their wellbeing, or an aggregator for mental health issues, depending on the religious attitudes adopted (Krok, 2015).

The understanding of wellbeing in post-primary education in Ireland is holistic and multi-faceted. Within the Irish post-primary context, it is recognised that “student wellbeing is present when students realise their abilities, take care of their physical wellbeing, can cope with the normal stresses of life, and have a sense of purpose and belonging to a wider community” (National Council for Curriculum and Assessment [NCCA], 2021, p. 13). As per the Junior Cycle Wellbeing Guidelines (NCCA, 2021), ‘wellbeing’ is used as a broad, umbrella term, which encompasses a number of facets within it: physical, social, emotional, intellectual, environmental and physical wellbeing. Delving deeper into this multi-faceted conceptualisation of wellbeing, it is understood that students must develop key skills and practice elements of ‘staying well’ in order to maintain their own wellbeing. These include: being physically healthy and active; being social: responsible use of technology; being safe; being positive about learning; being confident; and being spiritual. The guidelines also acknowledge the important role of the various aspects of a young person’s environment, as well as the relationships between these aspects, as influencing student wellbeing at post-primary level (NCCA, 2021).

Within the Irish context, recent review papers, as well as state literature on wellbeing, acknowledge the potential of religion to impact student wellbeing (Meehan, 2020; NCCA, 2017). Ireland’s Senior Cycle programme is the curriculum completed by students in their final two/three years of post-primary/second-level school; reforms to this programme are imminent (Department of Education [DE], 2022). In addition, wellbeing is an area of national priority in terms of education in Ireland (Department of Education and Skills [DES], 2018; NCCA, 2009, 2019, 2023). However, research which investigates the relationship between mental wellbeing and religion amongst students attending post-primary schools is limited within the Irish context. Further, such research is considered to be particularly pertinent in Ireland currently, given the upcoming reforms to the Senior Cycle programme, the vast changes in the religious landscape, as well as the recent decline in adolescents’ mental health in Ireland (Dooley and Fitzgerald, 2012; Dooley et al., 2019).

Therefore, this study aimed to address the gap present in the literature and to enhance understanding of the relationship between mental wellbeing and religion amongst adolescent students attending post-primary/second-level schools in Ireland. Previous studies in the international context have employed solely quantitative methodologies to investigate the relationships between psychological wellbeing and religion amongst adolescents (Wong et al., 2006). The current study aimed to capture the voices of adolescents in a more broad, holistic manner, through use of semi-structured interviews. With a small sample size of seven adolescents, the findings of this study are generalisable primarily to the individual adolescents who took part and point towards areas for further research.

## Key Constructs

### Mental Wellbeing

Definitions of the terms ‘mental health’ and ‘wellbeing’ have varied throughout the past century, with researchers and practitioners struggling to decide conclusively on a definite meaning for either term. Historically, the term ‘mental health’ was conceptualised as the mere absence of a mental illness, with a gradual move towards the consideration of mental health as a state of being (Bertolote, 2008). The WHO’s (2005) recent and widely-used definition insinuates that mental health refers to a “state of well-being” (p. 19) relative to the mind. ‘Wellbeing’, then, can be most simply defined as the state of ‘being well’ (Appleby, 2016); however, wellbeing is more holistic than mental health, and encompasses a broad array of components which contribute towards an individual’s overall sense of wellbeing, including physical, emotional, social, psychological/mental and economic wellbeing (Centers for Disease Control and Prevention [CDC], 2018).

The conceptualisation of psychological wellbeing, or mental wellbeing, has been the subject of much debate amongst researchers from differing fields of study. It is generally recognised that two distinct philosophical viewpoints have informed the varying definitions of psychological/mental wellbeing: hedonism, which theorises that happiness and pleasure are the highest sources of good, and eudaimonism, which emphasises the pursuit of meaning in life (Gao & McLellan, 2018). Such researchers include Keyes (2002), who purported that mental health comprises of three facets, namely, psychological/mental wellbeing, social wellbeing and emotional wellbeing. Consequently, within the current paper, ‘mental wellbeing’ is understood from the eudaimonic stance, as a state of wellbeing relative to the mind of the individual.

There has been considerable diversity in the various aspects of wellbeing studied within previous research amongst adolescents which has focused on religion and wellbeing. Many studies have investigated religion and its relationship with emotional wellbeing, e.g. life satisfaction and happiness (Abdel-Khalek, 2011; Davis III & Kiang, 2016; Holder, Coleman, Krupa & Krupa, 2016). Other researchers have focused on religion and its links with aspects of social wellbeing amongst young people (e.g. Butler-Barnes, Martin, Hope, Copeland-Linder & Lawrence Scott, 2015). However, following a thorough review of the literature, it was noted that a dearth exists regarding the relationship between psychological wellbeing and religion amongst adolescents. For this reason, it was decided to focus on psychological/mental wellbeing in this study, rather than focusing on social/emotional wellbeing or on wellbeing as a broader concept.

### Religion

Researchers and practitioners have long been interested in the scientific study of religion. As early as the beginning of the twentieth century, James (1902) philosophised that religious experience is concerned primarily with the supernatural, and therefore is not accessible to scientific means of investigation. Others adopted a more reductionist view, with Freud (1930) famously treating religious beliefs as a form of neurosis and Jung (1960) believing that religion holds its roots primarily in the unconscious mind. Humanistic psychologists, such as Maslow (1954) and Rogers (1961), attributed religiousness to the process of self-actualisation, or the realisation of one’s potential as a human being. Further, Frankl (1962) believed that humans are ultimately motivated by the search for meaning in life, which is inextricably linked to religion. In more recent times, transpersonal psychologists have adopted a broader approach and have purported that religious phenomena provide a connection for individuals with a transcendental reality; additionally, they assert that certain cognitive, social, emotional and behavioural elements of individuals’ religious experience can indeed be investigated by modern cognitive and neuro sciences (Tart, 1992; Wilber, 1995).

#### Religiosity

Within the social sciences, attempts at studying individuals’ religious experiences have focused primarily on the cognitive, social, emotional and behavioural aspects of religion (Holdcroft, 2006). The word ‘religion’ has its origins in the Latin ‘relgare’, which translates as ‘bind together’ (Oxford Learner’s Dictionaries, 2020). Thus, one’s ‘religiosity’ denotes a bond, or a relationship, between themselves and a supernatural power (Cohen et al., 2012). It is generally recognised that religiosity is a complex and multi-dimensional construct, encompassing emotional, intellectual, behavioural and motivational facets. These include personal prayer, participation in the activities of a religious tradition, religious beliefs, and self-professed religious affiliation (Holdcroft, 2006). Religiosity has also been used interchangeably with the term ‘religiousness’; this suggests that there exists a spectrum, whereby individuals may have higher or lower levels of personal religiosity (Hackney & Sanders, 2003).

#### Religious Coping

According to Pargament, Koenig and Perez (2000), “It is not enough to know that the individual prays, attends church, or watches religious television”, but it is more important to understand how their religion helps them to “understand and deal with stressors” (p. 521). The term ‘religious coping’, then, aims to specify how individuals utilise religion in order to comprehend and deal with the events of daily life (Dein, 2018). It is widely recognised that religious coping methods can be either positive or negative in nature. Examples of positive religious coping mechanisms include religious forgiveness, reading scriptures or sacred texts for strength, maintaining a secure relationship with God, and spiritual/congregational support (Pargament et al., 2004). Conversely, negative religious coping methods are considered to be maladaptive; examples of negative religious coping mechanisms include anger at or feeling abandoned by God and interpreting negative life events as a consequence of divine punishment (Hebert et al., 2009).

### Aim of the Current Study

Underpinned by the aforementioned theoretical perspectives on mental wellbeing, religion, and the systemic influence of religion on adolescents, this study sought to qualitatively explore the relationship between mental wellbeing and religion amongst a sample of adolescent students attending post-primary/second-level schools in Ireland.

## Method

### Research Design

The current paper reports on the qualitative aspect of a mixed-methods study. Online, individual semi-structured interviews were utilised to explore the perceptions of adolescent participants regarding the relationship between mental wellbeing and religion.

### Participants

As previously outlined, the qualitative data within the current paper were gathered as part of a mixed-methods study. In total, 14 participants (11 females and 3 males) who had partaken in the quantitative aspect of the study indicated their interest in becoming involved in the qualitative element of the research by providing contact details in a relevant section at the end of the quantitative survey. Each prospective participant was then contacted by the researcher on a first-come first-served basis, with an invitation to partake in a semi-structured interview on the research topic. Semi-structured interviews were conducted with 7 participants in total (5 females and 2 males), as the remainder of participants invited did not respond to the invitation to partake in the interview; additionally, it was deemed that data saturation had been obtained by the seventh interview (Boddy, 2016; Guest, Bunce and Kasparson, 2006). Each participant in the qualitative aspect of the study was assigned a pseudonym in order to protect their identity. Participants ranged in age between 15 and 19 years and attended post-primary schools within the Republic of Ireland. All students were completing the Senior Cycle programme and were in their fourth, fifth (penultimate) or sixth (final) year of post-primary school. As participants self-selected to partake in the interviews, demographic information was not factored into the selection of participants. Participant demographic characteristics are presented in Table 1.

**Table 1:** Participant demographic information

Pseudonym	Gender	Religious affiliation/Non-affiliation
Róisín	Female	Catholic
Kaspar	Male	Atheist
Niamh	Female	Catholic
Stacy	Female	Catholic
Clodagh	Female	Catholic
Seán	Male	Catholic
Molly	Female	Catholic

### Measures

#### Semi-Structured Interviews

Individual, semi-structured interviews were employed in order to obtain the viewpoints of participants in relation to mental wellbeing and religion and their perceptions regarding the potential relationships between them. Semi-structured interviews were deemed an appropriate choice of measure as they are compatible with a variety of methods of data analysis, including analysis evolving from the critical realist perspective (Smith and Elger, 2012). In the interest of participant confidentiality, it was decided to conduct these semi-structured interviews on a one-to-one basis with the researcher, due to the potentially personal and sensitive nature of the interview content.

As outlined above, previous research in the area has utilised solely quantitative methodologies to investigate the relationship between mental wellbeing and religion amongst adolescents. Therefore, it was necessary to devise a novel interview schedule for use within the current study. As the interview schedule utilised in this study had never previously been used, it was decided to pilot the schedule in order to ensure its usefulness and, subsequently, the credibility of the results of the present study (Merriam & Tisdell, 2015). Thus, the interview schedule was piloted in March 2021 with two individuals (one male and one female). Both participants in the pilot study provided oral feedback on the interview schedule at the end of the interview. Following this process, a number of minor amendments regarding the ordering of questions and use of language on the original schedule were made, resulting in the final interview schedule which was used in the present study. Piloting of the interview schedule was deemed to be an important aspect of the procedure in the current research in terms of including participants’ voices and preferences in a relational manner within the research methodology, in order to co-construct knowledge with participants, rather than viewing them as research subjects (Gergen, 2009; Gergen & Gill, 2020). Please see Appendix A for the final interview schedule used in this study.

#### Data Collection

Institutional ethical approval for the study was obtained prior to the commencement of data collection. Due to the Covid-19-related restrictions and school closures which were in place in Ireland at the time, data were collected solely online between February 2021 and May 2021. Adolescent participants completed an individual, online interview with the researcher using Zoom video-calling software, which was arranged at a suitable time for the participant. The interviews lasted between five and 20 min, depending on the level of detail the participant wished to share. All participants engaged with the each question asked during the interviews, with some participants offering more detailed and lengthier responses than others.

Written consent/assent for partaking in the semi-structured interviews was obtained in advance from participants and, where appropriate, their parents, via signed consent and assent forms. Verbal consent was also obtained from participants at the beginning of the interview, and participants were also reminded of their right to withdraw from the study, as well as the limits of confidentiality. With the consent of the participants, each interview was recorded using an audio recording device and transcribed verbatim immediately after the interview, in order to facilitate data analysis. A debriefing report was forwarded to participants via email after the culmination of the interview, along with a copy of their interview transcript, for cross-checking purposes. Confirmability of the results was aided through use of an audit trail, whereby the researcher kept a detailed record of the processes of data collection, analysis and interpretation (Korstjens & Moser, 2018).

### Data Analysis

**Fig. 1:**
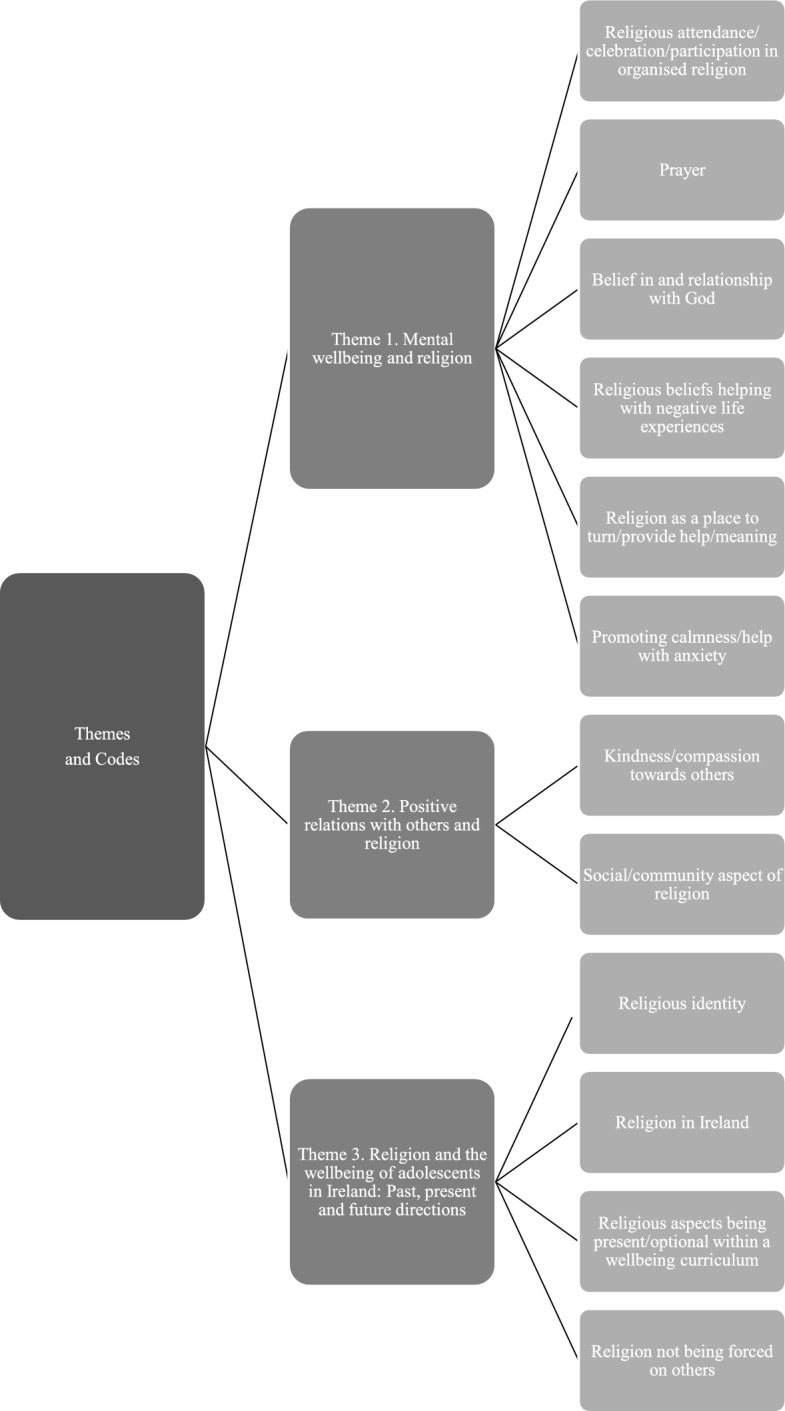
Themes and associated codes from thematic analysis

Qualitative data gathered from the semi-structured interviews were analysed using reflexive thematic analysis from the critical realist perspective (Braun & Clarke, 2019). Reflexive thematic analysis is an approach to qualitative data analysis which focuses primarily on the identification of themes, otherwise known as patterns of meaning, within the data. This approach to thematic analysis involves a recursive, six-phase process, which includes familiarising oneself with the data, coding, generating themes, reviewing themes, defining themes and reporting (Braun & Clarke, 2021). The results of the reflexive thematic analysis are discussed below under three headings, or themes, which arose from this analysis. Figure 1 presents the main themes and associated codes utilised when generating the themes. Pseudonyms are used when making reference to the participant who provided a response, for contextualisation.

#### Theme 1 – Mental Wellbeing and Religion

A number of aspects of religiosity and/or religious coping methods were mentioned by participants as being beneficial for their mental wellbeing, including holding religious beliefs, personal prayer and participation in organised religion. Positive religious coping methods mentioned by participants included the following: having a relationship with God, their religious beliefs helping them to cope with negative life experiences, and having religion as something to turn to in times of need.

Firstly, participants cited holding religious beliefs as being beneficial for their overall mental wellbeing. Molly felt that being aware of one’s religious beliefs was crucial for individuals’ mental wellbeing: “I think you can’t have a good mental wellbeing, a good mental health, without being grounded in your own faith and knowing what you believe in, and why you believe in it”. She also mentioned that her belief in God “really helps” to improve her “mental health and wellbeing”. Similarly, in speaking about the influence of her religious beliefs on her life, Róisín stated, “If I ever have a problem, like, if it was coming up to exams and I feel like I need a little bit of help, I can just, like…it’s always there…I kind of feel like someone is looking after me, I guess”.

The link between personal prayer and a perceived sense of calmness and alleviation of nervousness or anxiety was mentioned by a number of participants. According to Róisín, “I can just say the Rosary and, like, it kind of calms me down in a sense, that I’m not as worried”. In agreement, Stacy stated, “I think it kind of helps me be more relaxed. Praying kind of takes some anxieties away”. When asked if she believed her religiosity had an impact on her mental wellbeing, Molly replied affirmatively:

Yeah, definitely…it kind of helps me stay grounded and, you know, not get really flustered or overwhelmed…I think praying is the main thing… the fact that I feel like someone is hearing my problems and potentially they could be answered, or helped … God comes into it… I think that really helps with my mental health and wellbeing…if I’m having a very stressful day, or just kind of feeling a bit overwhelmed, I can just kind of sit down and relax and pray.

Participation in organised religion was another aspect of religiosity which was cited as being beneficial for mental wellbeing. In this regard, Seán, who identified as a Catholic, spoke about the benefits of receiving the sacrament of Confession, stating that this experience is “really helpful to get things off my chest and clear my conscience”.

One participant, Kaspar, who identified as an atheist, did not feel that this had an impact on his mental wellbeing. When asked whether he thought that being an atheist had an impact on his mental wellbeing, he stated, “No, uh…no, I don’t. No”.

Clodagh cited her relationship with God as being of great importance to her mental wellbeing, “I think, yeah, just having a relationship with God…I think that’s really helpful”. Seán reported that his mental wellbeing benefitted from “a connection with God…because…you feel that…you may be, that you have something special and that you are someone special to God, and that’s really helpful”. Similarly, Stacy related:

I think it definitely helps me with, like, experiences. Like, if I’m nervous about something, or if something’s kind of going on … it’s like a person that’s there that isn’t going to … that doesn’t necessarily have to answer or judge me, I can just kind of say these things and feel like they’re in the hands of someone else.

Some participants mentioned that their religious beliefs enabled them to make sense of experiences in their lives, and viewed this as valuable in terms of promoting their mental wellbeing. Niamh reflected on this in relation to how her religious beliefs help her to cope:

If something, like, bad happens, I have a feeling that…I know that it’s happening for a reason. That…bad things don’t just happen for no good reason, that God is there and that He has a plan, and that something even worse could be happening.

Additionally, Seán expressed how his beliefs have helped him to cope with negative events in his life, by allowing him to “explain some of the things I’m going through, through faith-based stuff”. He also noted the benefits of reading scripture, stating that he found reading the Bible “refreshing” and that it is has helped him to appreciate how “people get help from the Church and religion”. Further, Stacy shared her experience of how her religion and belief in the afterlife helped her to cope during adverse life events, including the Covid-19-related lockdowns and a family bereavement:

…in our family, my granddad wasn’t very well and he had passed away and I think with all of that, without religion, I don’t think that I would have been able to cope with that, because I just think that the with whole lockdown, and then when you lose someone … I think without religion and without being able to, kind of, have God as this person to come to, and be able to believe that my granddad is gone somewhere better, like, I don’t think I would have been able to cope without religion. Yeah. It definitely gets me through the tough times.

Religion was also cited by a number of other participants as being something they could turn to in times of need. Róisín explained the role of this in her life: “It’s something I feel I can always turn to…in tough times…if I’m not always in a good place, I have that place *to* go”. When asked if she felt her religion had an influence on her mental wellbeing, Clodagh responded by stating, “I think having something to, sort of, hold onto … when times are a bit hard, really helps…something to kind of, give you a bit of guidance and a path to go through”.

Notably, the religious coping mechanisms identified by participants as being helpful for their mental wellbeing would be considered as positive religious coping methods (Pargament et al., 1998), with no negative religious coping methods mentioned by any of the participants.

#### Theme 2 – Positive Relations with Others and Religion

The link between religion and compassion and kindness towards others was emphasised by a number of participants. Niamh stated that her religion increases her tendency to be “more compassionate with people”. Further, Seán noted that people who are religious often demonstrate kindness towards other people, stating, “not only is it many of the people in the community, but also many of the priests in my local parish are very helpful and kind”. Further, Stacy stated that her religion, and particularly her participation in a religious youth group, helps her to remember and live by “core beliefs”, such as “treat others like you want to be treated”.

The social aspect of organised religious activities and membership of a religious community was also cited by participants as having a positive impact on mental wellbeing. Stacy mentioned that being part of a religious youth group has “definitely helped” to improve her mental wellbeing. Similarly, Molly’s involvement in her religious community aided her helped her to “make friends”, which she stated was “good for my wellbeing”. Additionally, Seán felt that being part of a religious community aided his mental wellbeing, “because you’re able to meet others who’ve gone through the same things as you and you’re able to talk about it”. He also stressed that the members of his religious community and, in particular, the parish priests “have great connections with many of the people, and they talk and get involved with the community and the school, so that’s really nice and helps people’s wellbeing”.

#### Theme 3 – Religion and the Wellbeing of Adolescents in Ireland: Past, Present and Future Directions

The final theme which was generated from the data pertained to the role of religion in the wellbeing of adolescents in Ireland in the past and the present, as well as possible future directions for the role of religion in relation to young people’s wellbeing in the Irish context.

As part of the interview, participants were asked if they thought that religion had a role to play in the mental wellbeing of other young people in Ireland. From their responses, it was apparent that many participants were acutely aware of Ireland’s history as a country in which many people took religion quite seriously; however, participants had varying views on the place of religion in Ireland today. Niamh felt that Ireland is still a vastly “religious country”, whilst Róisín thought that it is “not really as common anymore for young people to practise their religion”. Kaspar, who identified as an atheist, mentioned that some of his friends “would go down to Mass and things”; however, he felt that religion no longer has a role to play in the mental wellbeing of young people in Ireland, as he did not think that “young people believe in it that much anymore”. Róisín was of the opinion that “if you do believe, it should help” to improve wellbeing. Similarly, Molly said, “I know plenty of people…a lot of my friends actually, who would say the same as me, that religion has a massive role in their lives, in … moving them forward, and also as part of their identity”, aligning with the PWB facet of autonomy.

In relation to future directions for religion and wellbeing in the lives of adolescents in Ireland, all adolescents who were religiously affiliated felt that there was a place for religion, or at least reference to certain aspects of religion, within a new wellbeing curriculum at Senior Cycle. According to Róisín, it would be important for a wellbeing curriculum to mention PRC strategies, such as “prayer, and what you can say to help yourself, and just…like, talking about the different places you can turn to, like, whether that’s going to light a candle, or kneeling down and saying the Rosary”. Additionally, Niamh thought that “talking about getting in touch with God” could be included, while Molly mentioned that “speaking about your feelings…like you do in religion when you’re praying, I think that would be a very nice thing to have in a wellbeing class”.

However, all participants unanimously felt that if these aspects of religion were to be included in a wellbeing class, that they should be included for students who are religious alone. Stacy mentioned that she attended a Catholic school, but that some students in her school “aren’t so religious” and that such religious elements within a wellbeing lesson could “maybe put some people off”. She instead felt that religious aspects could be included as an “add-in” part a wellbeing curriculum for pupils who are religious. Similarly, Kaspar, who identified as an atheist, thought that there could be “a different (wellbeing) class” for religious pupils to attend.

## Discussion

The current study qualitatively explored the relationship between mental wellbeing and religion amongst a sample of adolescent students attending post-primary/second-level schools in Ireland. The results of the reflexive thematic analysis demonstrated a range of positive outcomes for adolescents’ mental wellbeing arising from their religion.

Religiously-affiliated adolescent participants reported that aspects of their own personal religiosity had a range of benefits for their mental wellbeing. Specifically, participants mentioned that a positive and secure personal relationship with God was beneficial for their mental wellbeing. Participants felt that they had a presence looking after them and guiding and helping them in their lives, as well as a non-judgemental person to whom they could turn to with various problems they were experiencing. This findings mirrors that of previous international research, whereby having a relationship with God was associated with greater overall psychological wellbeing for adolescents (Butler-Barnes et al., 2018).

Other religious beliefs were also employed by participants to effectively cope with negative life events, such as a bereavement; this included for one participant a belief in the afterlife. These results correspond with those of previous studies, which have shown the potential for religious beliefs to be deployed positively in coping with life stressors (Pargament et al., 1992). In addition, personal reading of scripture was noted as being beneficial for a participant’s wellbeing as it helped him to understand how religion has helped in the lives of others, aligning with the findings of previous research in the area (Thomas & Barbato, 2020).

Engaging in individual personal prayer was beneficial for participants’ mental wellbeing, in that adolescents felt helped this practice alleviated feelings of nervousness, being overwhelmed, and anxiety. Prayer was utilised effectively by participants to cope during stressful times, for example prior to examinations; this corroborates the results of previous studies, which have shown that positive religious coping methods, such as seeking a connection with God through personal prayer, are beneficial for individuals who are dealing with stressful events (Krok, 2015). In addition, participation in organised religious activities at the individual level, including receiving the sacrament of Confession, was also mentioned as having positive implications for mental wellbeing, with benefits in terms of alleviation of feelings of guilt. This aligns with the views of international researchers, who suggest that participation in the sacrament of Confession for Catholics can alleviate certain types of guilt in a way that can be more effective than modern-day psychotherapy (Martinez-Pilkington, 2007).

One participant, who identified as an atheist, did not mention any aspects of religiosity as being beneficial or non-beneficial for his mental wellbeing. This suggests that religion can have benefits for the mental wellbeing of students, but only for those who hold and internalise their personal religious beliefs and connections and engage willingly in individual religious practices. This corresponds with similar hypotheses which have been forwarded by researchers in the international context (Wenger, 2011).

The findings of the current study may help to inform adolescents’ own understanding of the potential influence of religion on their mental wellbeing, with particular relevance to adolescents who are religiously affiliated. As a result, adolescents may be able to consider whether their deployment of religion in their lives has a positive or negative impact on their mental wellbeing, and adapt the manner of this deployment, if necessary.

Religion was mentioned by adolescents as being beneficial in terms of promoting more positive and ethical human interactions: for example, by encouraging kindness, helpfulness and compassion in the treatment of others. Participants reported that their treatment of others was influenced by the core values and standards of their religion. This corroborates the findings of previous research, which has shown that religious adolescents are more likely to engage in prosocial activities, such as helping others, volunteering, and other means of civic engagement (Donahue & Benson, 1995).

Participation in a religious youth group was noted by participants in the current study as being beneficial in terms of promoting mental wellbeing, through enhanced friendships with others and a greater sense of social connectedness as a result of being part of such a group. Similar findings have been reported from an international perspective (Smith, Webber and DeFrain, 2013). These findings may prove useful for adults who work with adolescents in both school and religious community settings. Adults involved in these communities should note the potential for adolescents’ religiosity and practice of positive religious coping methods to support positive mental wellbeing and, thus, to engage adolescents in practising elements of personal religiosity and positive religious coping methods, e.g., personal prayer, developing a relationship with God, and reading of sacred scripture for strength, as part of the activities of school or religious communities/youth groups.

### Limitations and Directions for Future Research

The most obvious limitation affecting this study is the small sample size and, thus, it cannot be generalised beyond the specific participant cohort. Nevertheless, it is an exploratory study which will hopefully encourage future research in this area. Within the current study, it is possible that the sampling strategy employed led to a certain degree of self-selection bias in the results (Jager et al., 2017), as adolescents who were more interested in religion, or who had positive views on the relationship between mental wellbeing and religion, may have been more likely to volunteer to participate in the study. This bias was particularly apparent in the religious demographics of the participants within the study, as six out of the seven participants who were interviewed were practising Catholics, who held largely positive views on the relationship between religion and their mental wellbeing. This reduces the likelihood of the results of the qualitative aspect of this study being applicable to pupils who are not religiously affiliated, to students who are affiliated to other religions, or to students who may have potentially negative views on the relationship between mental wellbeing and religion. Further, with a small sample size of seven adolescents, the findings of this study are generalisable primarily to the individual adolescents who took part.

As previously noted, the interview schedule devised for the study was novel and piloted in advance, as previous research in the area employed solely quantitative methods. However, improvements could be made to this schedule should it be used to guide future research in the area. For example, participants were not asked to articulate their understanding of religion, and as a result it was difficult to ascertain at certain times whether the participant made differentiations between religion as a subject, as a personal practice, or as a community practice. Similarly, participants were not asked to articulate their understanding of mental wellbeing during the interview, meaning that participants could have had differing understandings of this concept and whether or how it related to religion.

These limitations regarding sampling, measures and potential selection bias point towards areas for further investigation. Future research should, therefore, aim to recruit more participants from a variety of faith backgrounds and none, to ascertain their perspectives on the relationship between mental wellbeing and religion. In addition, future research should aim to capture the voices of students who have alternative views on the relationship between mental wellbeing and religion, particularly students who practice negative religious coping methods. Finally, the interview schedule should be reviewed and revised prior to use in future research; specifically, future researchers should aim to include questions at the outset which ask participants to clarify their understanding of the key concepts.

## Conclusions

This research explored the relationship between mental wellbeing and religion amongst a cohort of students at Senior Cycle level attending post-primary schools in Ireland, encompassing a holistic approach to capturing the voices of adolescents through use of qualitative measures. Overall, the findings demonstrated that when used positively, elements of personal religiosity and the practice of positive religious coping methods has the potential to positively impact and support the mental wellbeing of students across various systemic levels. While there are limitations associated with this study, the current research adds a distinct contribution in terms of advancing knowledge and understanding of the relationship between mental wellbeing and religion amongst adolescents within the Irish context, with various implications arising from the findings for adolescents themselves, for curriculum, as well as for adults who work with religious adolescents across both school and community settings.

## Appendix

### Appendix A: Interview Schedule for Semi-Structured Interviews

Script: “Thank you for agreeing to engage in an interview about your views on religion and mental wellbeing with me today. There are no right or wrong answers to any of the questions, I would just like to hear your own views on these topics. If there is a question which you do not feel comfortable answering, please feel free to let me know and we can continue on to the next question. You can stop or pause this interview at any time, without giving any reason. Everything that you say today will be kept completely confidential and will be in no way identifiable to you. The only exceptions to this are if you tell me that you are being harmed by someone, if you tell me that you are harming someone, or if you tell me that you are in danger of harming yourself. Is that okay? Do you have any questions before we begin?”.

1.Are you part of a religious denomination? (If so) In what ways do you practise your faith?

2.In what ways does your religion influence your life?/In what ways does religion influence your life, if any?

3.Do you think your religion has an influence on your mental wellbeing? (If so) In what ways does it influence your mental wellbeing?

4.Do you think religion has a role to play in the mental wellbeing of (other) young people in Ireland? Why/why not?

5.If ‘Wellbeing’ was introduced as a subject in school, would you like to see religious elements included as part of this subject? Why/why not? (If yes – What kind of things could be included?).

6. Do you have any other comments?
